# Ultrasound-Guided Hydrodilatation With Triamcinolone Acetonide for Adhesive Capsulitis: A Randomized Controlled Trial Comparing the Posterior Glenohumeral Recess and the Rotator Cuff Interval Approaches

**DOI:** 10.3389/fphar.2021.686139

**Published:** 2021-05-07

**Authors:** Jia-Chi Wang, Po-Yi Tsai, Po-Cheng Hsu, Jian-Ru Huang, Kevin A. Wang, Chen-Liang Chou, Ke-Vin Chang

**Affiliations:** ^1^ Department of Physical Medicine and Rehabilitation, Taipei Veterans General Hospital, Taipei, Taiwan; ^2^ School of Medicine, National Yang-Ming University, Taipei, Taiwan; ^3^ School of Medicine, National Yang Ming Chiao Tung University, Taipei, Taiwan; ^4^ Divison of General Surgery, Department of Surgery, Shin-Kong Memorial Hospital, Taipei, Taiwan; ^5^ School of Medicine, Fu Jen Catholic University, New Taipei City, Taiwan; ^6^ Department of Physical Medicine and Rehabilitation and Community and Geriatric Research Center, National Taiwan University Hospital, Bei-Hu Branch and National Taiwan University College of Medicine, Taipei, Taiwan; ^7^ Center for Regional Anesthesia and Pain Medicine, Wang-Fang Hospital, Taipei Medical University, Taipei, Taiwan

**Keywords:** adhesive capsulitis, hydrodilatation, corticosteroid, ultrasound, rotator cuff interval

## Abstract

For patients with adhesive capsulitis, hydrodilatation is typically performed using corticosteroids with ultrasound guidance via the posterior glenohumeral recess. Recently, a new intervention technique via the rotator cuff interval has been described. This study aimed to compare the efficacy of hydrodilatation with triamcinolone acetonide via the posterior glenohumeral recess and the rotator cuff interval in patients with adhesive capsulitis. This prospective randomized controlled trial was conducted in a tertiary care center with a follow-up period of 12 weeks. We enrolled 64 patients diagnosed with shoulder adhesive capsulitis. The subjects were randomly assigned to two groups that received hydrodilatation with corticosteroids either through the posterior glenohumeral recess or though the rotator cuff interval. The injection contained 4 ml of triamcinolone acetonide (40 mg) mixed with 4 ml of 2% lidocaine hydrochloride and 12 ml of normal saline. The shoulder pain and disability index, visual analog scale for pain, and range of motion were analyzed before and at 6 and 12 weeks after the treatment. Both groups experienced improvements in the visual analog scale scores, shoulder pain and disability index scores, and range of motion throughout the study period. A significant group-time interaction was observed in terms of the visual analog scale for pain during motion (*p* = 0.019), favoring hydrodilatation through the rotator cuff interval. Thus, hydrodilatation through the rotator cuff interval might be a better treatment option than that through the posterior glenohumeral recess for patients with adhesive capsulitis, considering its superior effect in alleviating pain during shoulder movement.

## Introduction

Adhesive capsulitis (AC), i.e., painful stiff shoulders, is characterized by progressive painful limitation of the shoulder motion, resulting in disability and impaired quality of life (Neviaser, 1987; Porcellini et al., 2013). The prevalence of AC ranges from 2 to 5% in the general population and is predominant in women, especially at 40–60 years of age (Zreik et al., 2016). Although the etiology of primary AC remains unknown, the idiopathic inflammation of the synovium and capsule of the glenohumeral joint is postulated to be the leading cause (Hand et al., 2007).

As AC is primarily considered an inflammatory disease (Hand et al., 2007), intra-articular corticosteroid injections have been frequently used for its treatment. Glenohumeral joint hydrodilatation with the administration of corticosteroids, another therapeutic approach, was first introduced by Andren and Lundberg (1965). The mechanism of hydrodilatation is based on the expansion of the joint cavity through the hydraulic pressure of the injectate administered in the capsule. The mechanical effect of hydrodilatation, in addition to decreased intra-articular inflammation after corticosteroid injection, was proven to improve the clinical outcomes of AC in at least three meta-analyses (Wu et al., 2017; Lin et al., 2018; Saltychev et al., 2018).

Hydrodilatation is typically performed using ultrasound guidance via the posterior glenohumeral joint. Recently, a new injection technique via the rotator interval was described (Juel et al., 2013; Ricci et al., 2020). The rotator cuff interval is a triangular area in the anterosuperior aspect of the shoulder bordered by the supraspinatus and subscapularis muscles and the coracoid process (Petchprapa et al., 2010). It contains the long head of the biceps tendon, coracohumeral ligament, superior glenohumeral ligament, and anterosuperior portion of the glenohumeral capsule. An antecedent arthroscopic study revealed that the predominant pathology in AC could be found near the rotator cuff interval, revealing extensive synovitis and proliferation of the vascular granulation tissue (Wiley, 1991). The approach targeting the rotator cuff interval is theoretically more effective than targeting the glenohumeral joint.

This study aimed to compare the short-(6 weeks) and intermediate-term (12 weeks) effects of hydrodilatation with triamcinolone acetonide through the posterior glenohumeral recess and through the rotator cuff interval. We hypothesized that the rotator cuff interval approach would achieve greater reduction in pain and better improvement in function as compared to the posterior glenohumeral recess approach.

## Materials and Methods

### Design

This was a prospective randomized control trial in compliance with the Consolidated Standards of Reporting Trials statement. The study was approved by the Institutional Review Board of Taipei Veteran General Hospital, Taiwan, and was registered at ClinicalTrials.gov (ClinicalTrials.gov Identifier: NCT03678038). All participants provided written informed consent and were randomly assigned to two groups undergoing hydrodilatation either though the posterior glenohumeral recess or though the rotator cuff interval, respectively. Randomization was performed by a computer using a random sequence generator in a block of four without stratification. The number of patients in each group was the same. Concealment of treatment assignment was maintained in sealed envelopes, which were opened only before the interventions. This study used an open-label design, because the participants would know their group allocation through the differences in the area of injection.

### Participants

Participants were recruited from the outpatient clinics of the Department of Physical Medicine and Rehabilitation at the Taipei Veterans General Hospital. The diagnosis of AC was based on clinical history, physical examination, and ultrasonography and radiography findings.

The inclusion criteria were as follows: 1) age 35–65 years (to prevent the inclusion of patients with secondary AC), 2) onset of shoulder stiffness since over a month, and 3) limitation in the passive range of motion (ROM) over 30° when compared with the contralateral side in at least two of these three movements: forward flexion, abduction, or external rotation.

The exclusion criteria were as follows: 1) ultrasound findings of rotator cuff tears, 2) plain radiography findings of significant glenohumeral joint arthritis, 3) accompanying cervical radiculopathy, 4) systemic inflammatory joint disease, 5) intra-articular injection into the glenohumeral joint within the past 3 months, 6) history of surgery on the affected shoulders, 7) regular use of systemic non-steroidal anti-inflammatory drugs or corticosteroids, and 8) allergy to corticosteroid or lidocaine.

### Intervention

All patients received one shot of ultrasound-guided hydrodilatation by a single physiatrist, using an Acuson S2000 machine (Siemens Healthcare, Erlangen, Germany) with a linear 9–12 MHz probe. The injection fluid contained 4 ml of 40 mg (10 mg/ml) triamcinolone acetonide (Shincort) mixed with 4 ml of 2% lidocaine hydrochloride (xylocaine) and 12 ml of normal saline.

**FIGURE 1:**
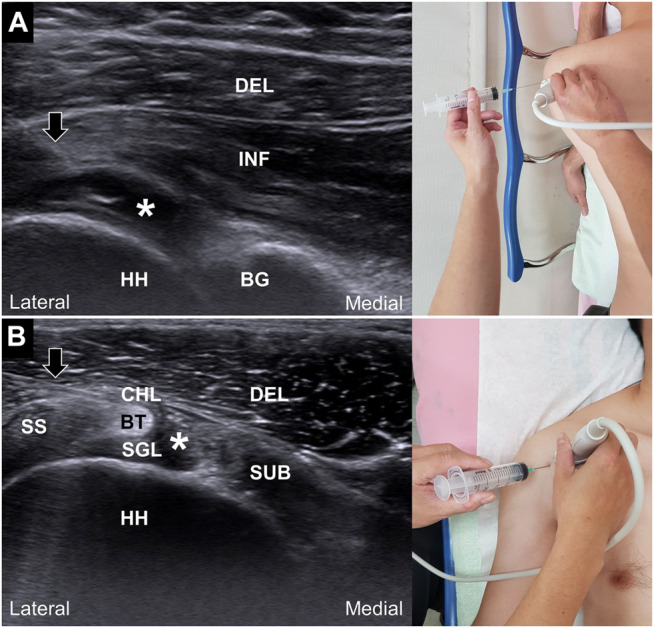
Ultrasound guided hydrodilatation through the posterior glenohumeral recess **(A)** and rotator cuff interval **(B)**. Black arrows: needles; white asterisks: injectate; DEL: deltoid muscle; INF, infraspinatus muscle; HH, humeral head; BG, bony glenoid of the scapulae; SS, supraspinatus tendon; SUB, subscapularis tendon; CHL, coracohumeral ligament; BT, long head of the biceps tendon; SGL, superior glenohumeral ligament.

In the group receiving hydrodilatation through the posterior glenohumeral recess, the patients were positioned side-lying with the injected shoulder on the top and the ipsilateral arm resting on a pillow (Figure 1). The probe was placed parallel to the lateral end of the scapular spine. After sterile preparation, a 23-gauge, 2.75-inch needle was inserted using the in-plane approach from the lateral side of the probe to target the articular junction between the humeral head and the bony glenoid fossa. The injectate was gently distributed into the posterior glenohumeral joint; a successful injection was indicated by recognition of gradual capsular distension.

In the group receiving hydrodilatation through the rotator cuff interval, the patients were placed in the supine position with the shoulder in slight abduction and extension to facilitate the visualization of the rotator cuff interval. The probe was placed lateral to the coracoid process on the deltopectoral groove. After sterile preparation, a 22-gauge, 1.75-inch needle was inserted using an in-plane approach from the lateral side of the probe. The needle was inserted deep into the coracohumeral ligament to reach the sheath of the long head of the biceps tendon (Figure 1). The injectate was slowly introduced into the peritendinous area in order to visualize the gradual distension of the glenohumeral capsule.

All the injection procedures were conducted by the same physician, who had more than 8 years of experience in ultrasound-guided injection techniques. We prescribed acetaminophen for our enrolled patients after the injections. They were advised to take the medication within the dosage of allowance when the visual analogue scale (VAS) of pain exceeded 4/10. Before the injection, a leaflet describing the post-injection exercise program was handed to the participants and the program was instructed by the same instructor. The program consisted of Codman's exercise, wall climbing exercise, shoulder external and internal rotation exercise using the bar and towel stretching exercise behind the back. All patients started the home-based exercises at the first day after injections. They were advised to perform the exercises for a minimum of two sessions per day. During the study period (within 12 weeks after interventions), the participants were allowed to receive physical therapy at our outpatient clinics. The modalities used during physical therapy comprised hot packing, ultrasound diathermy and inferential current therapy.

### Outcome Measurements

The primary outcome was the shoulder pain and disability index (SPADI) score. The secondary outcomes were the VAS score for pain and ROM of the shoulder. All assessments were performed by a research assistant, who was blinded to the treatment allocation, at baseline and at 6 and 12 weeks after the treatment.

The SPADI was used to assess the severity of pain and disability. A previous study has shown that the Chinese version of the SPADI has high internal consistency and test-retest reliability (Yao et al., 2017). It consists of 13 items that are divided into 2 subscales: pain scale (5 items) and disability scale (8 items) (Roach et al., 1991). Each item is rated from 0 (no pain/no difficulty) to 10 (worst pain experienced/very difficult). The score is then transformed to a 100-point scale, with the highest score indicating the most severe pain and disability. In the literature, the minimal and clinically relevant difference for SPADI has been reported to be 10 points (Williams et al., 1995).

Regarding VAS, patients were asked to indicate the intensity of their average level of pain in the affected shoulder within the past 1 week using an 11-point scale, ranging from 0 (no pain) to 10 (worst pain imaginable).

In terms of ROM, the degrees of shoulder flexion, abduction, external and internal rotation were measured by a goniometer in the supine position in a random sequence. External and internal rotation was measured with the shoulder in 90° of abduction and the elbow in flexion of 90°. If shoulder abduction was less than 90°, external and internal rotation was measured with the shoulder in the maximal degree of abduction. During follow-up, the shoulder was positioned at the same degree of abduction to measure the ROM of external and internal rotation. Each ROM was measured twice and the average values were used for analysis.

### Statistical Analysis

Based on an antecedent randomized controlled trial comparing the effect of different injection methods for AC (Sun et al., 2018), our sample size was designed to detect a standardized mean difference of 0.3 in the shoulder function change between both the groups. The standard deviation was set at 0.5. Assuming a drop-out rate of 20%, an alpha level (α) of 0.05, and a power (β) of 80%, the estimated sample size was 56.

The Shapiro-Wilk test was used to determine whether the continuous variables were normally distributed. In the case of normal distribution, univariate analysis of the continuous variables was conducted using one-way analysis of variance, or else the Mann-Whitney U-test was used. The chi-square test was used for comparison of the categorical data. If data with small cell counts were encountered, the Fisher’s exact test was used. The effect size of the between-group difference was denoted by Cohen’s d, derived from the between-group mean difference divided by the pooled standard deviation.

Repeated measurement analysis of variance was employed to analyze the effect of group-time interaction, which was considered the outcome of interest. The within-subjects factor was the time points of outcome measurements (baseline and 6 and 12 weeks following interventions). The difference in the treatment approaches (hydrodilatation through the posterior glenohumeral recess vs. hydrodilatation through the rotator cuff interval) was regarded as the between-subjects factor. Repeated measurement analysis of variance was also used to compare the continuous variables within the same group across different time points, using the Bonferroni test for post-hoc analysis. The intention-to-treat principle was used in this study. Regarding the missing data due to loss of follow-up, we handled this issue by using the method of last observation carried over (Pampaka et al., 2016). The last observations were defined the last observations prior to dropout for those lost to follow-up. The analyses were implemented using MedCalc 14.0 (MedCalc Software, Ostend, Belgium) statistical software; *p*-values <0.05 were considered statistically significant.

## Results

### Screening, Enrollment, and Lost to Follow-Up

**FIGURE 2:**
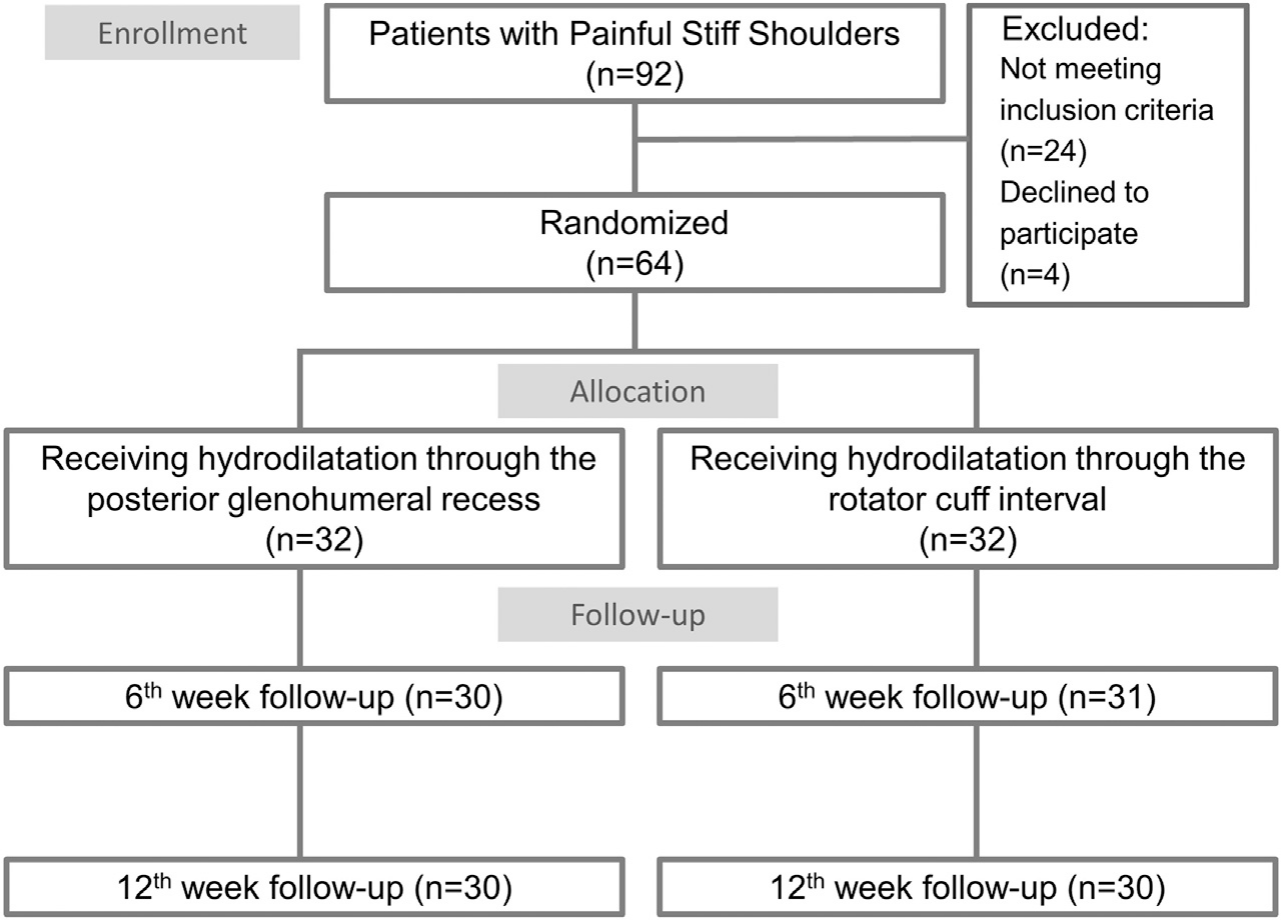
Study flow diagram for the groups receiving ultrasound guided hydrodilatation through the posterior glenohumeral recess or through the rotator cuff interval.

In total, 92 participants, who met the inclusion criteria, visited our clinic. Of these, 64 patients participated in the trial after providing informed consent and were equally randomized into the group undergoing hydrodilatation through the posterior glenohumeral recess (*n* = 32) and that undergoing hydrodilatation through the rotator cuff interval (*n* = 32). Three patients did not visit the follow-up clinic in the 6th week, whereas four patients were lost to follow-up in the 12th week (Figure 2). There was no significant difference in the proportion of patients lost to follow-up between the two groups (*p* = 1.00). The baseline characteristics, including age, sex, body weight, body height, SPADI scores, VAS scores for pain (at rest, at night, and during motion), and shoulder ROM, were similar across both the groups (Table 1).

**TABLE 1:** Baseline characteristics of the patients.

	Group 1 (*N* = 32)	Group 2 (*N* = 32)	*p* Value of between-group comparisons
Age (years)	53.96 ± 7.02 (51.43–56.50)	52.40 ± 6.37 (50.10–54.70)	0.355
Female (number, %)	19 (59.37%)	20 (62.50%)	0.798
Height (cm)	162.74 ± 7.01 (159.71–165.78)	161.25 ± 6.98 (158.30–164.20)	0.469
Weight (kg)	63.96 ± 11.18 (58.87–69.06)	59.16 ± 10.60 (54.33–63.99)	0.161
Body mass index (kg/m^2^)	24.07 ± 3.13 (22.65–25.50)	22.88 ± 3.82 (21.14–24.62)	0.276
Right shoulder (number, %)	16 (50.00%)	12 (37.50%)	0.313
Diabetes mellitus (number, %)	7 (21.87%)	5 (15.62%)	0.522
Pain duration (month, mean ± SD)	6.65 ± 4.10 (5.17–8.13)	6.12 ± 3.15 (4.98–7.26)	0.564
SPADI (pain)	46.87 ± 15.48 (41.29–52.45)	50.62 ± 13.28 (45.83–55.41)	0.302
SPADI (function)	49.57 ± 22.07 (41.61–57.52)	47.65 ± 14.98 (42.25–53.05)	0.686
SPADI (total)	48.62 ± 16.62 (42.63–54.62)	48.79 ± 12.00 (44.47–53.12)	0.963
VAS at rest	2.50 ± 2.25 (1.68–3.31)	1.62 ± 2.04 (0.88–2.36)	0.087
VAS at night	5.25 ± 2.87 (4.21–6.28)	4.65 ± 2.81 (3.64–5.67)	0.598
VAS during motion	5.15 ± 2.15 (4.37–5.93)	5.90 ± 2.29 (5.08–6.73)	0.122
Abduction (°)	89.43 ± 21.40 (81.72–97.15)	91.75 ± 22.81 (83.52–99.97)	0.871
External rotation (°)	18.21 ± 25.74 (8.93–27.50)	19.59 ± 28.39 (9.35–29.83)	0.797
Internal rotation (°)	28.18 ± 30.04 (17.35–39.02)	23.12 ± 28.35 (12.90–33.34)	0.451
Flexion (°)	127.12 ± 17.44 (120.83–133.41)	131.12 ± 19.25 (124.18–138.06)	0.387

* indicates *p* < 0.05. The values of continuous variables were expressed by the mean and standard deviation (SD). The values of categorical variables were expressed by the number (percentage). Group 1 indicates hydrodilatation though the posterior glenohumeral recess, whereas Group 2 indicates hydrodilatation through the rotator cuff interval.

### Outcome Measurements

**FIGURE 3:**
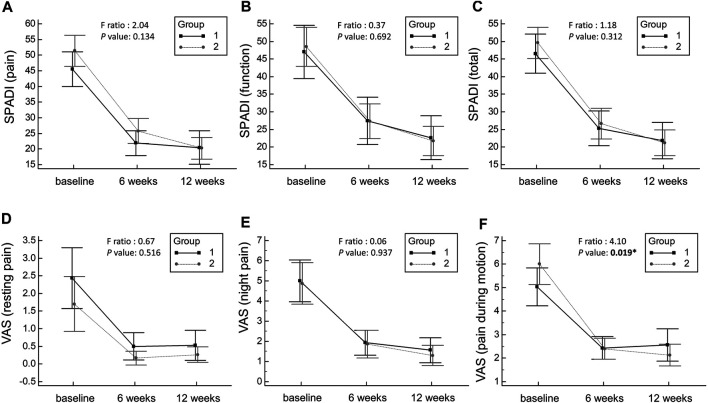
Mean changes and the corresponding 95% confidence intervals pertaining to the pain domain of the SPADI scores **(A)**, the function domain of the SPADI score **(B)**, the total SPADI scores **(C)**, the VAS scores at rest **(D)**, the VAS scores at night **(E)** and the VAS scores during motion **(F)**. SPADI: Shoulder Pain and Disability Index; VAS, visual analogue scale. Group 1 indicates hydrodilatation though the posterior glenohumeral recess, whereas Group 2 indicates hydrodilatation through the rotator cuff interval.

**FIGURE 4:**
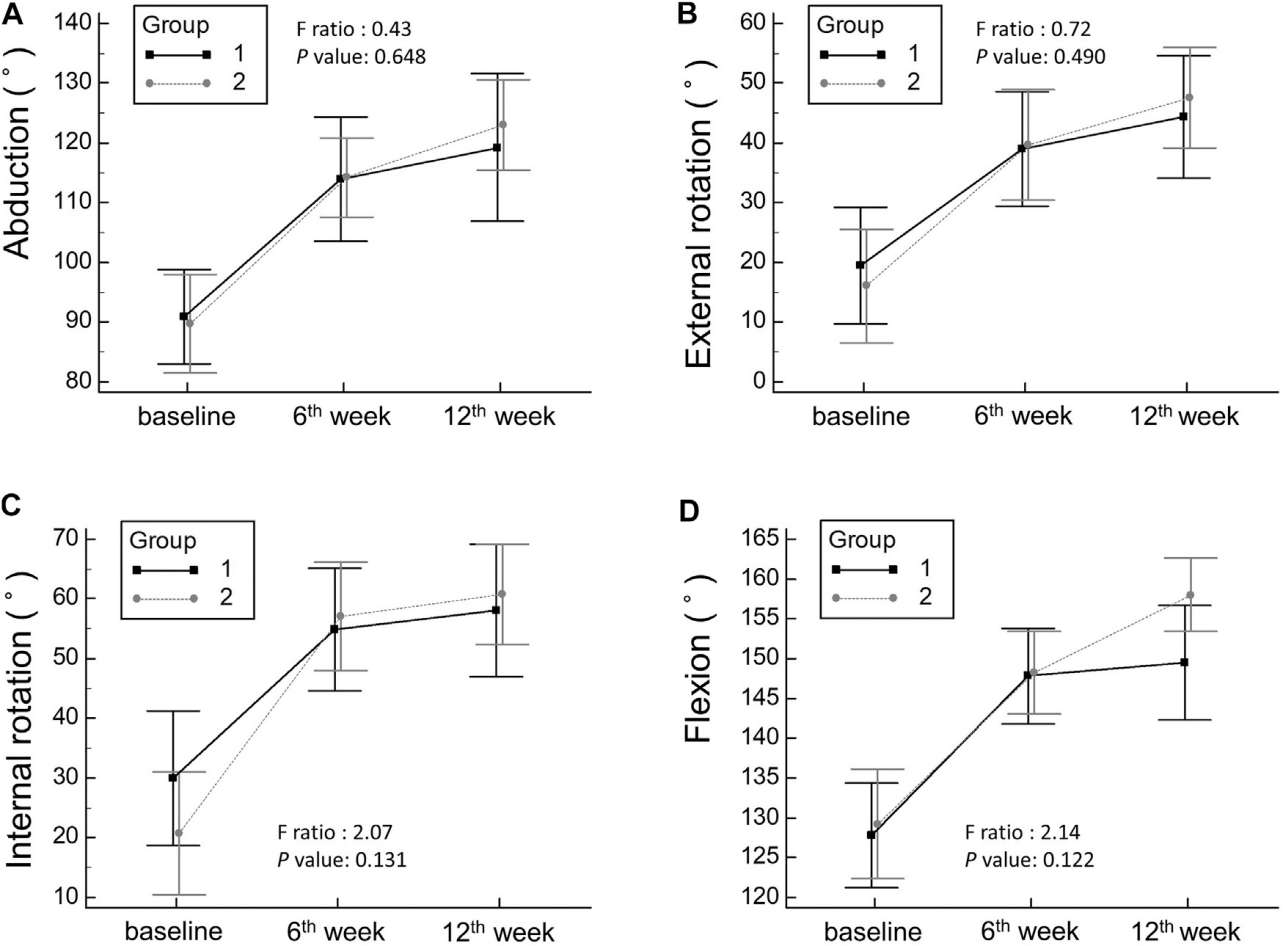
Mean changes and the corresponding 95% confidence intervals pertaining to shoulder range of motion in abduction **(A)**, external rotation **(B)**, internal rotation **(C)**, and flexion **(D)**. Group 1 indicates hydrodilatation though the posterior glenohumeral recess, whereas Group 2 indicates hydrodilatation through the rotator cuff interval.

A significant decrease in the SPADI scores (pain, function, and total) (Table 2; Figure 3) and in the VAS scores for pain (at rest, at night, and during motion) (Table 3; Figure 3) and an increase in the shoulder ROM in all directions (Table 4; Figure 4) were observed in the 6th week of follow-up after injection in both groups. The improvement remained constant or progressed in the 12th week of follow-up in all aspects of shoulder symptoms and functional measurements. There was a greater reduction in the VAS scores of pain during motion between the baseline and 6th week and between the baseline and 12th week in the group receiving hydrodilatation through the rotator cuff interval as compared to the group undergoing hydrodilatation through the posterior glenohumeral recess (Table 3). No significant group-time interaction was identified regarding the individual domains and total SPADI scores, VAS scores for pain at rest and at night, and shoulder ROM in all directions. A significant group-time interaction was observed in terms of VAS for pain during motion (*p* = 0.019). The F-ratio and *p*-value for all the repeated measurements are presented in Figures 3, 4, respectively.

**TABLE 2:** Shoulder Pain and Disability Index (SPADI) scores at the baseline, 6th week and 12th week after the injection.

	Group 1 (*n* = 32)	Difference from baseline	Group 2 (*n* = 32)	Difference from baseline	*p* Value	Cohen’s *d*
SPADI (pain)						
Baseline	46.87 ± 15.48^a,b^ (41.29–52.45)	-	50.62 ± 13.28^a,b^ (45.83–55.41)	-	-	-
6th week	23.83 ± 15.08^a^ (18.30–29.37)	−22.54 ± 14.10 (−27.72 to −17.37)	25.76 ± 10.60^a,c^ (21.80–29.72)	−25.63 ± 11.96 (−30.09 to −21.16)	0.361	0.240
12th week	20.46 ± 14.23^b^ (15.15–25.78)	−25.00 ± 13.85 (−30.17 to −19.82)	20.26 ± 9.34^b,c^ (16.77–23.75)	−31.13 ± 12.85 (−35.92 to −26.33)	0.081	0.467
SPADI (function)						
Baseline	49.57 ± 22.07^a,b^ (41.61–57.52)	-	47.65 ± 14.98^a,b^ (42.25–53.05)	-	-	-
6th week	28.95 ± 19.58^a,c^ (21.76–36.13)	−19.27 ± 11.23 (−23.39 to -15.15)	27.29 ± 13.16^a,c^ (22.37–32.20)	−21.16 ± 12.41 (−25.80 to −16.53)	0.675	0.149
12th week	22.66 ± 16.53^b,c^ (16.49–28.84)	−24.33 ± 13.04 (−29.20 to −19.46)	21.75 ± 11.03^b,c^ (17.62–25.87)	−26.70 ± 12.10 (−31.22 to −22.18)	0.468	0.192
SPADI (total)						
Baseline	48.62 ± 16.62^a,b^ (42.63–54.62)	-	48.79 ± 12.00^a,b^ (44.47–53.12)	-	-	-
6th week	27.02 ± 16.09^a^ (21.11–32.92)	−20.59 ± 10.19 (−24.33 to −16.85)	26.69 ± 11.71^a,c^ (22.31–31.06)	−22.89 ± 9.89 (−26.59 to −19.20)	0.302	0.219
12th week	21.81 ± 13.72^b^ (16.69–26.94)	−24.69 ± 10.80 (−28.72 to −20.65)	21.17 ± 9.74^b,c^ (17.54–24.81)	−28.41 ± 9.86 (−32.09 to −24.72)	0.169	0.366

Scores are given as mean ± standard deviation (95% confidence interval of mean). *p* values pertain to between-group comparisons for differences from baseline. * indicates *p* < 0.05. Group 1 indicates hydrodilatation though the posterior glenohumeral recess, whereas Group 2 indicates hydrodilatation through the rotator cuff interval. SPADI (total) = [SPADI (pain) *0.5 + SPADI (function)*0.8]*(10/13).

a significant difference between the baseline and 6th weeks in the same group.

b significant difference between the baseline and 12th week in the same group.

c significant difference between the 6th and 12th weeks in the same group.

**TABLE 3:** Visual analogue scale of pain scores at the baseline, 6th weeks and 12th weeks after the injection.

	Group 1 (*n* = 32)	Difference from baseline	Group 2 (*n* = 32)	Difference from baseline	*p* Value	Cohen’s *d*
Resting pain						
Baseline	2.50 ± 2.25^a,b^ (1.68–3.31)	-	1.62 ± 2.04^a,b^ (0.88–2.36)	-	-	-
6th week	0.64 ± 1.30^a^ (0.16–1.12)	−1.83 ± 1.96 (−2.56 to −1.11)	0.16 ± 0.53^a^ (−0.03–0.36)	−1.53 ± 1.85 (−2.22 to −0.84)	0.499	0.185
12th weeks	0.53 ± 1.13^b^ (0.10–0.95)	−1.90 ± 1.91 (−2.61 to −1.18)	0.26 ± 0.58^b^ (0.04–0.48)	−1.43 ± 2.11 (−2.22 to −0.64)	0.253	0.228
Night pain						
Baseline	5.25 ± 2.87^a,b^ (4.21–6.28)	-	4.65 ± 2.81^a,b^ (3.64–5.67)	-	-	-
6th week	2.09 ± 1.85^a^ (1.41–2.77)	−3.00 ± 1.98 (−3.72 to −2.27)	1.86 ± 1.83^a^ (1.18–2.55)	−3.00 ± 2.33 (−3.87 to −2.12)	1.000	<0.001
12th week	1.56 ± 1.65^b^ (0.94–2.18)	−3.43 ± 2.40 (−4.33 to −2.53)	1.30 ± 1.34^b^ (0.79–1.80)	−3.56 ± 2.83 (−4.62 to −2.50)	0.597	0.167
Pain during motion						
Baseline	5.15 ± 2.15^a,b^ (4.37–5.93)	-	5.90 ± 2.29^a,b^ (5.08–6.73)	-	-	-
6th week	2.64 ± 1.72^a^ (2.01–3.27)	−2.45 ± 2.20 (−3.25 to −1.64)	2.40 ± 1.19^a^ (1.95–2.84)	−3.60 ± 1.97 (−4.33 to −2.86)	0.017*	0.260
12th weeks	2.56 ± 1.85^b^(1.87–3.25)	−2.46 ± 2.16 (−3.27 to −1.65)	2.13 ± 1.22^b^ (1.67–2.59)	−3.86 ± 2.31 (−4.73 to −3.00)	0.019*	0.636

VAS scores are given as mean ± standard deviation (95% confidence interval of mean), *p* values pertain to between-group comparison for difference from baseline. * indicates *p* < 0.05. Group 1 indicates hydrodilatation though the posterior glenohumeral recess, whereas Group 2 indicates hydrodilatation through the rotator cuff interval.

a significant difference between baseline and 6 weeks in the same group.

b significant difference between baseline and 12 weeks in the same group.

c significant difference between 6 and 12 weeks in the same group.

**TABLE 4:** Abduction, external rotation, internal rotation and flexion degrees at the baseline, 6th week and 12th week after injection.

	Group 1 (*n* = 32)	Difference from baseline	Group 2 (*n* = 32)	Difference from baseline	*p* Value	Cohen’s *d*
Abduction						
Baseline	89.43 ± 21.40^a,b^ (81.72–97.15)	-	91.75 ± 22.81^a,b^ (83.52–99.97)	-	-	-
6th week	113.16 ± 27.73^a^ (102.98–123.33)	22.83 ± 21.63 (14.90–30.77)	114.23 ± 17.89^a,c^ (107.55–120.91)	24.46 ± 20.58 (16.77–32.15)	0.707	0.141
12th week	119.20 ± 33.15^b^ (106.81–131.58)	28.26 ± 27.77 (17.89–38.63)	123.06 ± 20.16^b,c^ (115.53–130.59)	33.30 ± 25.33 (23.83–42.76)	0.579	0.171
External rotation						
Baseline	18.21 ± 25.74^a,b^ (8.93–27.50)	-	19.59 ± 28.39^a,b^ (9.35–29.83)	-	-	-
6th week	37.74 ± 26.06^a^ (28.18–47.30)	18.93 ± 21.48 (11.05–26.81)	39.66 ± 24.70^a,c^ (30.44–48.88)	23.60 ± 19.56 (16.29–30.90)	0.379	0.231
12th week	44.36 ± 27.29^b^ (34.17–54.55)	24.93 ± 26.56 (15.01–34.85)	47.53 ± 22.47^b,c^ (39.14–55.92)	31.46 ± 27.32 (21.26–41.66)	0.352	0.247
Internal rotation						
Baseline	28.18 ± 30.04^a,b^ (17.35–39.02)	-	23.12 ± 28.35^a,b^ (12.90–33.34)	-	-	-
6th week	53.12 ± 28.68^a^ (42.60–63.65)	24.03 ± 28.84 (13.45–34.61)	57.03 ± 24.16^a^ (48.00–66.05)	36.30 ± 29.34 (25.34–47.25)	0.062	0.254
12th week	58.10 ± 29.82^b^ (46.96–69.23)	28.03 ± 31.62 (16.22–39.84)	60.76 ± 22.67^b^ (52.29–69.23)	40.03 ± 30.93 (28.48–51.58)	0.143	0.390
Flexion						
Baseline	127.12 ± 17.44^a,b^ (120.83–133.41)	-	131.12 ± 19.25^a,b^ (124.18–138.06)	-	-	-
6th week	147.64 ± 15.88^a^ (141.81–153.47)	19.87 ± 16.17 (13.93–25.80)	148.26 ± 13.81^a,c^ (143.10–153.42)	19.03 ± 19.19 (11.86–26.20)	0.457	0.193
12th week	149.50 ± 19.28^b^ (142.29–156.70)	21.70 ± 18.59 (14.75–28.64)	158.00 ± 12.33^b,c^ (153.39–162.60)	28.76 ± 18.85 (21.72–35.80)	0.149	0.384

The values are given as mean ± standard deviation (95% confidence interval of mean). *p* values pertain to between-group comparisons for differences from baseline. * indicates *p* < 0.05. Group 1 indicates hydrodilatation though the posterior glenohumeral recess, whereas Group 2 indicates hydrodilatation through the rotator cuff interval.

a significant difference between baseline and the 6 weeks in the same group.

b significant difference between baseline and the 12 weeks in the same group.

c significant difference between the 6 and 12 weeks in the same group.

### Adverse Events

The procedure was well tolerated by the patients in both approaches, and no serious adverse events were observed. Two patients (one in each group) reported significant post-injection pain (VAS score >4) on the first day after the intervention, which resolved spontaneously without the need for additional treatments.

## Discussion

The present randomized controlled trial aimed to compare the effectiveness of hydrodilatation with triamcinolone acetonide through the posterior glenohumeral recess and through the rotator cuff interval. Our results revealed that both the approaches contributed to significant improvement in the shoulder pain and function for at least 3 months in the patients with AC. A significant group-time interaction was observed in terms of VAS during motion, favoring hydrodilatation through the rotator cuff interval approach.

To date, only one trial has investigated the influence of the injection sites of hydrodilatation on AC. Elnady et al. (2020) conducted a controlled trial of 60 patients who were randomized to either ultrasound-guided hydrodilatation with corticosteroid (40 mg methylprednisolone acetate, 1 ml 2% lidocaine, and 15 ml saline) via the posterior glenohumeral recess or the rotator cuff interval. All patients received one injection and were evaluated 3 months post-treatment. The group undergoing hydrodilatation via the rotator cuff interval showed significantly greater improvement in the VAS scores, SPADI scores, and ROM (flexion, abduction, and external rotation) as compared to the group undergoing hydrodilatation via the posterior glenohumeral recess. In our study, there was a greater reduction in the VAS score for pain during motion between baseline and 6th week and between baseline and 12th week in the group treated by hydrodilatation through the rotator cuff interval as compared to the group undergoing hydrodilatation through the posterior glenohumeral recess. However, our results revealed no significant group-time interaction regarding SPADI scores, VAS scores for pain at rest and at night, and shoulder ROM in all directions.

AC is thought to begin with inflammatory hypervascular synovitis, which elicits a progressive fibroblastic change in the adjacent capsule (Hand et al., 2007). Increased expression of inflammatory cytokines has been observed in the synovial tissue of patients with AC (Tamai et al., 2014). By injecting corticosteroids into the glenohumeral joints, the synovial inflammation can be suppressed through the reduction of the inflammatory cytokines, thus contributing to pain relief and improvement of the shoulder function. Hydrodilatation with corticosteroids has gained popularity owing to its theoretical benefit of simultaneous management of synovial inflammation and capsular fibrosis. The commonly used route for hydrodilatation is through the posterior glenohumeral recess. Saltychev et al. reviewed the efficacy of hydrodilatation based on 12 trials and concluded that combined hydrodilatation with corticosteroid injection distension provides the best pain relief and recovery of the ROM limitation in patients with AC (Saltychev et al., 2018). The reviews conducted by Lin et al. and Wu et al. showed that combined hydrodilatation with corticosteroid injection provided better recovery of the ROM in the external rotation in patients with AC (Wu et al., 2017; Lin et al., 2018).

In recent studies, the rotator cuff interval has been implicated as the key structure in the pathogenesis of AC (Ozaki et al., 1989; Neer et al., 1992; Tamai et al., 2014). The first component usually affected is the coracohumeral ligament. Neer et al. proposed that a tight coracohumeral ligament might limit the external rotation of the arm, which can be seen early in AC patients (Neer et al., 1992). Two prospective, single-arm clinical trials supported the administration of ultrasound-guided injection through the rotator cuff interval for managing AC (Juel et al., 2013; Yoong et al., 2015). Juel et al. demonstrated that corticosteroid injection (20 mg of triamcinolone and 3 ml of 2% lidocaine) in the rotator cuff interval could improve the pain, SPADI scores, and ROM at the 4- and 12-weeks follow-up (Juel et al., 2013). Yoong et al. evaluated corticosteroid hydrodilatation (40 mg of triamcinolone, 10 ml of 1% lidocaine, and 10 ml of 0.5% bupivacaine) and found that there was complete resolution of the symptoms in 32% of the patients and satisfactory improvement in 54% of the patients at the 4-months follow-up (Yoong et al., 2015).

In this study, hydrodilatation through the rotator cuff interval was more effective in improving the pain during motion as compared to hydrodilatation through the posterior glenohumeral recess. Compared with the measurements of pain at rest or at night, pain during motion is more sensitive in evaluating the progress of AC because the disease is characterized by motion limitation. These results are comparable with those reported by Elnady et al. (2020). There are two possible reasons for these findings. First, the injection of corticosteroids through the rotator cuff interval modified the inflammatory reaction near the coracohumeral ligament, which plays a key role in the development of AC. Second, the therapeutic efficacy of intra-articular corticosteroid injection differs according to the anatomy of the joint cavity (Kim et al., 2015). In our experience, hydrodilatation through the rotator cuff interval provided greater distension of the subscapular recess than that through the posterior glenohumeral recess. The difference in the distribution of the injectate according to the injection site could be a possible cause of pain relief during motion after intervention through the rotator cuff interval. Furthermore, because the subacromial-subdeltoid bursa is located superficial to the rotator cuff interval, some injection material might be distributed into the adjacent bursae during hydrodilatation through the rotator cuff interval. Subacromial steroid injection has been reported to be effective in reducing the pain and recovering shoulder function in patients with AC (Shin and Lee, 2013; Sun et al., 2018). Simultaneous injection into the glenohumeral joint and subacromial bursa during hydrodilatation in the rotator cuff interval could explain its superiority in relieving pain during motion as compared to the posterior glenohumeral recess approach. Further studies are warranted to explore the distribution of the injectate during hydrodilatation through the rotator cuff interval. In addition, injection through the rotator cuff interval might facilitate distribution of the injectate to juxta-articular space. The terminal branches of the axillary and lateral pectoral nerves would be infiltrated, leading to a better analgesic effect (Yamak Altinpulluk et al., 2020a; Yamak Altinpulluk et al., 2020b; Galluccio et al., 2020; González-Arnay et al., 2020).

However, unlike the study by Elnady et al. (2020), hydrodilatation though the rotator cuff interval did not achieve better recovery in the shoulder function and ROM as compared to the posterior glenohumeral recess approach in our study. These discrepancies might be due to the discrepancy in the disease stages between our study and Elnady et al.‘s study (Elnady et al., 2020). The mean interval from symptom onset to treatment in our participants was 6.3 months (range, 1–12 months). In the study by Elnady et al., patients had symptoms for less than 6 months; in comparison, our participants presented with longer disease durations and possibly more fibrotic and stiffer joints, leading to reduced ROM improvement following hydrodilatation. In addition, during hydrodilatation through the rotator cuff interval, the injection material may leak into the subacromial bursa. Thus, the injected fluid might not be completely distributed into the glenohumeral space to distend the joint capsule sufficiently. Leakage of the fluid into the subacromial bursa could be a possible reason for the lack of significant differences in the shoulder function and ROM improvement between the two groups. Furthermore, in our experience, due to the contracted and thickened coracohumeral ligament, the resistance against injection in the rotator cuff interval is high. The abovementioned factor might increase the procedure time if the physician is not familiar with this technique. Thus, we suggest that the addition of ultrasound-guided fenestration to release the coracohumeral ligament in future treatment protocols to improve the effectiveness of ROM recovery. Additionally, a prospective study might be needed hereafter to compare the procedure time between the approaches through the rotator cuff interval and posterior glenohumeral recess.

The optimal volume of distension during hydrodilatation remains unclear. The injection volume varied significantly among the prior studies (Wu et al., 2017). Two approaches have been described, including the capsule-rupturing and capsule-preserving hydrodilatation methods (Kim et al., 2011; Lee et al., 2017). In the study done by Kim et al. (2011), the capsule-preserving technique provided greater improvement than the capsule-rupturing technique for treating AC. In our study, without using a real-time pressure monitoring device, we were not able to know whether the capsule was ruptured or not. However, the average volume to achieve capsule rupture ranged between 18 and 25 ml based on the research performed by Lee et al. (2008), implying that there was a likely high proportion of our patients with preserved capsules after injections of 20 ml of fluid.

Hydrodilatation through the rotator cuff interval has several advantages over hydrodilatation through the posterior glenohumeral recess. First, parts of the injectate infiltrate into the subacromial bursa, which contributes to better pain control if the patients have concomitant subacromial impingement. Second, it is easier to assess the rotator cuff and subacromial bursa before the procedure to prevent unwanted corticosteroid injections in those who already have rotator cuff tendon tears. Third, in patients with a larger body size, the approach through the posterior glenohumeral recess would be more challenging than the method through the rotator cuff interval using the linear transducer.

In our study, the primary outcome was the between-group difference of the changes in the total SPADI score. Based on our data, the change of the total SPADI score from baseline to the 12th week was −24.69 ± 10.80 in the group using the posterior-glenohumeral-recess approach and −28.41 ± 9.86 in the group using the rotator-cuff-interval approach. To recognize a mean difference of 3.72 with a standard deviation of 10.34 in the SPADI score, a total of 246 participants were required for reaching a power (β) of 0.8 and an alpha (α) level of 0.05. Therefore, even though the actual number of the enrolled participants exceeded the estimated sample size by around 14%, the small difference in the SPADI score was not able to be recognized. Furthermore, a previous study (Roy et al., 2009) suggested the minimal clinically important difference for the SPADI score ranged between 8 and 13 points, which was larger than the between-group difference (3.72) of ours. Therefore, based on our data, we were able to claim that no significant difference in improvement of shoulder function between both approaches for patients with AC.

### Study Limitations and Strength

Our study has some limitations. First, the follow-up period was only 12 weeks; therefore, the long-term effects of the treatment remain unknown. Further research should incorporate a longer study period to examine the long-term effects of hydrodilatation through the rotator cuff interval for treating AC. Second, we included subjects with AC at different stages. The disease duration recalled by the participants might not be accurate, and the precise stage of the AC could not be determined, which might have affected our results. Third, due to the design of the intervention, the participants could not be blinded to the intervention protocol. Fourth, the study did not include a placebo group; the improvement of the outcomes in both the groups might be partially attributed to the natural recovery of the disease. However, as the effectiveness of hydrodilatation with corticosteroid has been proven to be comparable to or even better than corticosteroid injection (Wu et al., 2017), the inclusion of a placebo group may be less ethical. Fifth, we did not request the participants to keep an exercise diary to track their exercise frequency and duration. Although the participants were encouraged to do home-based exercises twice per day, the documents of their compliance were not available. Therefore, a subgroup analysis based on the compliance of post-injection exercise could not be performed in the present study.

Despite these limitations, this study has some strength. First, this was an adequately powered randomized-controlled study with a relatively low rate of loss to follow-up. Second, all patients were required to have a radiograph, ultrasound, and clinical assessment by the same physician to ensure the accuracy of AC diagnosis. Third, all injections were performed by a single experienced physician with sonographic guidance to ensure that the injectate was injected inside the target region.

## Conclusion

Ultrasound-guided hydrodilatation with triamcinolone acetonide achieved better pain relief during motion through injection in the rotator cuff interval than through the posterior glenohumeral recess in patients with AC. Nevertheless, there was no significant difference in the shoulder function and ROM recovery between the two intervention methods. Therefore, hydrodilatation through the rotator cuff interval might be a better treatment option for patients with AC, considering its superior effect on alleviating pain during movement when compared with the posterior glenohumeral recess approach.

## Data Availability

The raw data supporting the conclusions of this article will be made available by the authors, without undue reservation.

## Abbreviations

AC, Adhesive capsulitis; SPADI, Shoulder Pain and Disability Index; VAS, Visual Analog Scale; ROM, Range of motion.
